# Mercury chloride alters heterochromatin domain organization and nucleolar activity in mouse liver

**DOI:** 10.1007/s00418-022-02151-8

**Published:** 2022-09-22

**Authors:** Lorena Zannino, Andrea Pagano, Claudio Casali, Monica Oldani, Alma Balestrazzi, Marco Biggiogera

**Affiliations:** 1 Department of Biology and Biotechnology ‘L. Spallanzani’, University of Pavia, 27100 Pavia, Italy; 2 Department of Biology and Biosciences, University of Milano-Bicocca, 20126 Milan, Italy

**Keywords:** Mercury Chloride, Cell stress, Epigenetics, Heterochromatin, Nucleolus, Ribogenesis

## Abstract

Mercury is a highly toxic element that induces severe alterations and a broad range of adverse effects on health. Its exposure is a global concern because it is widespread in the environment due to its multiple industrial, domestic, agricultural and medical usages. Among its various chemical forms, both humans and animals are mainly exposed to mercury chloride (HgCl_2_), methylmercury and elemental mercury. HgCl_2_ is metabolized primarily in the liver. We analysed the effects on the nuclear architecture of an increasing dosage of HgCl_2_ in mouse hepatocytes cell culture and in mouse liver, focusing specifically on the organization, on some epigenetic features of the heterochromatin domains and on the nucleolar morphology and activity. Through the combination of molecular and imaging approaches both at optical and electron microscopy, we show that mercury chloride induces modifications of the heterochromatin domains and a decrease of some histones post-translational modifications associated to heterochromatin. This is accompanied by an increase in nucleolar activity which is reflected by bigger nucleoli. We hypothesized that heterochromatin decondensation and nucleolar activation following mercury chloride exposure could be functional to express proteins necessary to counteract the harmful stimulus and reach a new equilibrium.

**Supplementary Information:**

The online version contains supplementary material available at 10.1007/s00418-022-02151-8.

## Introduction

Mercury represents one of the main agents responsible for environmental pollution. It is the third most dangerous heavy metal, after arsenic and lead, according to the Agency for Toxic Substance and Disease Registry (ATSDR) (Zhang et al. 2017)

We can come in contact with mercury in different contexts, since its big employment in many human activities has led to its wide distribution (Raghuvanshi et al. 2016; Altunkaynak et al. 2015).

Mercury is corrosive and once absorbed into the bloodstream, it can bind plasma proteins or enter the erythrocytes. It crosses the biological membranes and is metabolized in the liver, then it accumulates in the kidneys. Consequently, these are considered the main target organs for mercury damage (Berlin et al. 2015). Mercury has a strong affinity for endogenous biomolecules, it easily forms organo-mercury complexes with proteins (Joshi et al. 2017), especially those proteins, peptides and aminoacids containing thiol groups, leading to a profound deterioration of fundamental metabolic processes (Wiggers et al. 2008). In fact, HgCl_2_ liver accumulation induces oxidative stress through glutathione (GSH) depletion, and mitochondrial depolarization, because of interference with enzyme functions, disturbing ATP production and protein synthesis (Joshi et al. 2017; Agarwal 2010). Cell physiology and integrity, in general, are affected due to decreased GSH, leading to an increase of reactive oxygen species (ROS) such as superoxide anion radicals, hydrogen peroxide and hydroxyl radicals, which provoke lipid, protein, and DNA oxidation (Abarikwu et al. 2018). In addition, HgCl_2_ is a potent apoptosis inducer, through cytochrome c release, activation of p38 mitogen-activated protein kinases (MAPK) and increase in nuclear factor kappa-B (NF-κB) (Yang et al. 2016).

Nevertheless, its effects on the nucleus are less studied. Nuclear architecture and gene expression are profoundly influenced by external cues and stressful stimuli (Zannino et al. 2021). Chromatin organization in molecular structures with different degrees of packaging and condensation can be modulated according to gene transcription and especially through epigenetic modifications (Fedorova and Zink 2008).

Generally speaking, there are two different states of chromatin in the genome. Heterochromatin, which is characterized by a more compact structure, less accessible to transcription factors, and euchromatin, more relaxed and permissive to transcription. Heterochromatin can be further subdivided into constitutive and facultative. The former refers to heterochromatin domains that are permanently silenced, while the second defines regions more prone to be interconverted to euchromatin upon different stimulations. The trimethylation of histone H3 on lysine 9 and 27 (H3K9me3 and H3K27me3) and on histone H4 on lysine 20 (H4K20me3) are associated to chromatin condensation and found in the heterochromatin domains (Cui et al. 2019). On the other hand, trimethylation of histone H3 tail on different lysines can be found in the euchromatin, such as H3K4me3 which is associated with active transcription (Howe et al. 2017).

Another important index of nuclear activity is the nucleolus. Being the site of ribosome biogenesis, it primarily influences protein synthesis and consequently cell metabolism and cell ability to respond and counteract stressful injuries (Dubois and Boisvert 2016).

In this study, we analysed the effects of HgCl_2_ exposure on mouse hepatocytes cell culture and liver nuclei. Microscopy approaches and especially transmission electron microscopy have allowed looking inside the nucleoplasm to gain insights into chromatin organization with fine details. Moreover, immunocytochemistry at electron microscopy has the advantage to show protein localization with great precision. We exploited this powerful technique to investigate nuclear architecture and some epigenetic changes caused by mercury chloride.

Our morphological analysis showed an evident heterochromatin decondensation and an increase in the size of the nucleoli following treatment with mercury chloride. Therefore, we decided to evaluate whether the concentration of some epigenetic markers, whose role in the formation of heterochromatin is universally accepted, varied in their quantity and distribution, after mercury stimulation. Specifically, we considered two epigenetic modifications present in the constitutive heterochromatin: H3K9me3 and H4K20me3 (Nehmé et al. 2015) and one associated to the facultative heterochromatin H3K27me3 (Trojer and Reinberg 2007). This to evaluate if mercury chloride treatment may have a different impact on these markers present in regions of our genome that are differently silenced.

We then verified the possibility that mercury chloride could induce or limit the expression of the methyltransferases which mediate the establishment of these epigenetic modifications.

Kmt5b is the histone methyltransferase known to establish the mono-, di- and tri-methylation of lysine 20 of histone H4 to produce respectively monomethylated ‘Lys-20’ (H4K20me1), dimethylated ‘Lys-20’ (H4K20me2) and trimethylated ‘Lys-20’ (H4K20me3) that regulate transcription and maintenance of genome integrity. This methyltransferase mainly functions in pericentric heterochromatin regions, thereby playing a central role in the establishment of constitutive heterochromatin (Bromberg et al. 2017). Suv39h1 is the Histone methyltransferase that specifically trimethylates ‘Lys-9’ of histone H3, using monomethylated H3 ‘Lys-9’ as substrate. H3 ‘Lys-9’ trimethylation represents a specific tag for epigenetic transcriptional repression by recruiting HP1 (CBX1, CBX3 and/or CBX5) proteins to methylated histones (Lehnertz et al. 2003). EZH2 is a Polycomb group (PcG) protein, the catalytic subunit of the PRC2/EED-EZH2 complex, which methylates ‘Lys-27’ (H3K27me) of histone H3, leading to transcriptional repression of the affected target gene. It is able to mono-, di- and trimethylate ‘Lys-27’ of histone H3 to form H3K27me1, H3K27me2 and H3K27me3 respectively, but plays a major role in forming H3K27me3, which is required for embryonic stem cell identity and proper differentiation (Lanzuolo and Orlando 2012).

Moreover, we quantified the HPTM H3K4me3, which is found in the actively transcribed genes (Howe et al. 2017), to estimate if mercury chloride treatment could induce an increase of this marker in the hepatocyte nuclei and favor gene expression.

Secondly, having noticed an increase in the size of nucleoli by transmission electron microscopy, we measured nucleolar size on a larger number of cells at optical microscopy by silver staining of the Nucleolar organizer regions (NORs). NORs are chromosomal portions that form clusters of tandemly repeated sequences of ribosomal genes. In eukaryotes, each unit is composed of three genes coding for 18S, 5.8 and 28S ribosomal RNA (rRNA), separated by two intergenic spacers and an external transcribed spacer (Britton-Davidian et al. 2012). The NOR size measured by this technique can provide an estimation of nucleolar size and activity.

In addition, we quantified the expression of the rRNAs, some transcription factors involved in the rRNA processing and two ribosomal proteins part of the large and small ribosomal subunits, to provide information about the possible effects of mercury on different aspects of ribogenesis, that is the prominent activity of the nucleolus.

We analysed the expression of three transcription factors: ubtf, baz2a and rrp9. Ubtf recognizes the ribosomal RNA gene promoter and activates rRNA transcription mediated by RNA polymerase I through cooperative interactions with the transcription factor SL1/TIF-IB complex, by binding specifically to the upstream control element (UCE) (Bell et al. 1988). Baz2a is an essential component of the NoRC (nucleolar remodeling complex), a complex that mediates silencing of a fraction of rDNA by recruiting histone-modifying enzymes and DNA methyltransferases, leading to heterochromatin formation and transcriptional silencing (Zhou et al. 2002).

Finally, we monitored the expression of Rrp9 because it code for a component of a nucleolar small nuclear ribonucleoprotein particle (snoRNP), thought to participate in the processing and modification of pre-rRNA (Chen et al. 2016).

The different parameters measured influence at different level cell metabolism and activity which in turn define the degree of cell damage and cell response to damage.

## Materials and methods

### Cell culture and tissue hepatocytes exposure to the toxicant HgCl_2_ and sample preparation

AML12 mouse hepatocytes (ATCC Cat# CRL-2254, RRID:CVCL_0140) were cultured following the manufacturer’s instructions.

Hepatocytes were exposed to increasing doses of HgCl_2_: 1, 5 and 10 µM by adding it to the complete cell culture medium for 1 h, maintaining the cell according to their growth protocol at 37 °C with 5% CO_2_ in the air atmosphere. Cells were then harvested, centrifuged at 800 rpm for 10 min and fixed in 4% paraformaldehyde for 30 min at room temperature (R.T.) and for 1 h and 30 min at 4 °C. Then they were rinsed with Phosphate Saline Buffer (PBS) 1X. Afterward, cells were centrifuged at 1200 rpm for 10 min. Pellets were stored at − 80 °C for RNA extraction and reverse transcription—quantitative real‐time polymerase chain reaction (qRT-PCR) analysis or pre-embedded in agar for immunocytochemistry at transmission electron microscopy (TEM). Pre-embedding is necessary to avoid sample loss in the following steps of sample preparation, which are reported in the next paragraph –Mouse liver tissue- as the procedure for cells and tissue dehydration and embedding in acrylic resin for TEM are the same for both.

### Mouse liver tissue

Four 5-week-old female and 6-month-old male B6C3F1 mice (RRID:IMSR_JAX:100,010) were purchased from Charles River (Como, Italy). Animals were maintained under controlled room conditions (22 °C, with 60% air moisture, and 12L:12D photoperiod). The liver was isolated from the 3-month-old mouse of the resulting female F1 offspring. The liver was cut into small pieces and immersed in DMEM F12 culturing medium added with 10 µM HgCl_2_ (treated tissue) or without HgCl_2_ as untreated control for 1 h at 37 °C with 5% CO_2_.

Tissue samples were then fixed with 4% paraformaldehyde in PBS 1X pH 7.2–7.4 at 4 °C for 2 h, incubated 30 min in NH_4_Cl 0,5 M at 4 °C to block free aldehyde groups and rinsed 3 times using PBS 1X.

Physical dehydration with a graded ethanol series was performed before the embedding in LR White acrylic resin, tissue samples were then incubated for 30 min in 50% ethanol and 50% LR White. An overnight infiltration with LR White at 4 °C follows. Tissue samples were finally embedded in gelatine capsules and resin polymerization was carried out at a temperature of 60 °C for 24 h.

Thin sections of 70–80 nm were cut with an ultramicrotome and collected on formvar-carbon-coated nickel grids (300 Mesh) for TEM analysis.

As discussed in the introduction, as far as we know, there are very little data about HgCl_2_ toxicity in the cell nucleus. Time and doses of HgCl_2_ treatments were chosen based on the previous studies, where concentrations of HgCl_2_ above 10 µm were reported to induce extensive cell death in a short time (Nieminen et al. 1990) and even milder treatments (HgCl_2_ concentrations lower than 10 µM) were reported to have important effects on the mitochondria and cell metabolism (Palmeira and Madeira 1997).

### Ethics approval

The experiments of this study conducted on animals were performed following the guiding principles of European (n. 86/609/CEE) and Italian (n. 116/92, 8/94) laws protecting animals used for scientific research.

### Immunocytochemistry at transmission electron microscopy

The grids carrying samples ultrathin sections were floated on a drop of 2% normal goat serum (NGS) diluted in PBS 1X pH 7.2–7.4 for 5 min at R.T. and then incubated overnight at 4 °C with the primary antibody diluted in Tween20 0,05% /PBS 1X pH 7.2–7.4. The following day, after two rinses in tween20 0,05%/PBS 1X pH 7.2–7.4 and two rinses in PBS 1X pH 7.2–7.4, the incubation in NGS was repeated as in the first step, followed by incubation with secondary antibody conjugated with 12 nm colloidal gold grain for 30 min at R.T. Finally, sections were rinsed two times in PBS 1X pH 7.2–7.4 and two in distilled water (dH_2_O) and allowed to dry. As a negative control, the same experimental procedure was performed using an equal volume of Tween20 0,05% /PBS 1X pH 7.2–7.4 without the primary antibody. A counterstaining to increase the contrast of cell structures is required: sections were counterstained by Ethylenediaminetetraacetic acid disodium salt solution (EDTA) regressive technique described below.

The primary antibodies used for immunocytochemistry are listed in the table EMS_1 in the Supplementary information. The secondary antibody was 12 nm Colloidal Gold AffiniPure Goat Anti-Rabbit IgG (H + L) (EM Grade) from Jackson Immunoresearch Biotechnology West Grove, Pennsylvania. The secondary antibody was diluted 1:20 in PBS 1X pH 7.2–7.4.

Manufacturers have proven the specificity of the antibodies used in this study. Moreover in western blot analysis on hepatocytes protein extracts, the same antibodies recognized a correctly sized band for each specific protein. We also proved signal specificity of the secondary antibody on liver tissue and AML12 cell culture hepatocytes by omitting each primary antibody (data not shown, available upon request).

### EDTA regressive technique

Regressive staining is a technique used to counterstain sections in TEM which exploits EDTA as a chelating agent to reveal with heavy contrast cell structures containing RNA, while deoxyribonucleoproteins and heterochromatin lose most of the stain (Bernhard 1969). In detail, after a pre-staining with uranyl acetate 4% aqueous solution, the thin sections were briefly floated on EDTA 0.2 M and finally post-stained with lead citrate (Reynolds 1963). Then sections were then allowed to dry and finally visualized on JEM-1400 Flash JEOL electron microscope operating at 80 kV.

### HPTMs quantification and statistical analysis

The density of the antigen -Histone post-translational modification (HPTM)- of interest was calculated as follows: 100 squares of a definite heterochromatin area (100 nm^2^ for hepatocytes from cell culture and 200nm^2^ for liver tissue for the heterochromatin markers quantifications; 500nm^2^ for H3K4me3 quantification) were identified and the HPTM labeling, marked by gold grain and appearing as dark spots, per single area was quantified. The measurement was performed considering 10 cells for each sample and 10 squares per single cell. Statistical significance was estimated by unpaired Student’s *t* test for comparison of two groups. *P* < 0.05 was considered statistically significant.

### Osmium ammine staining

The osmium ammine staining is used in TEM for the specific detection of DNA. It is based on a Feulgen-type reaction, consisting of acid hydrolysis to obtain free aldehyde groups on DNA followed by their binding to osmium ammine, a Schiff-type reagent. Since the DNA interaction with the osmium ammine is specific, the nuclear domains characterized by high DNA concentration, like heterochromatin, appear dark on a lighter background (Biggiogera et al. 1996). The grids were laid on HCl 5 N solution for 30 min in a well. Then, 7 quick rinses in dH_2_O followed by 3 rinses, 2 min each, in dH_2_O were performed. Afterwards, the grids were laid on osmium ammine solution for 60 min and finally rinsed in dH_2_O to reduce precipitates formation as follows: 7 quick rinses, 3 rinses of 2 min each, 3 rinses of 5 min each, 1 rinse of 20 min. At the end of each rinse, the grids were blotted on absorbent paper. As a negative control, the reaction was also conducted by staining with osmium ammine, but avoiding the hydrolysis step with HCl 5 N.

For morphometric analysis, all specimens were observed with a Jeol JEM-1200EXIII electron microscope equipped with a 30 mm objective aperture and operating at 80 kV. Images were analysed using the software Fiji (RRID:SCR_002285). We analysed the mean grey intensity of 50 heterochromatin areas (100 nm^2^) in total from 10 different nuclei. Lower intensity (darker areas) should correspond to locally higher DNA concentration and therefore to heterochromatin regions that adopt a more condensed conformation.

### Nuclear staining with Hoechst 33258

Semi-thin sections about 500 nm in thickness from samples embedded in acrylic LR White resin, as described in details for TEM sample preparation, were cut using an ultramicrotome.

For Hoechst staining, sections were initially hydrated by 4 rinses in PBS 1X pH 7.2–7.4, 2 min each and incubated 5 min in Hoechst 33,258 (1ug/mL) in the dark. 4 rinses, 2 min each, followed by 1 rinse for 5 min in PBS 1X pH 7.2–7.4, were then performed. Glass slides were finally mounted using 90% glycerol in PBS 1X pH 7.2–7.4.

Hepatic tissue and cell sections were imaged using an Olympus BX51 microscope

Images were submitted to morphometric analyses using the software Fiji (RRID:SCR_002285).

### AgNOR staining

AML 12 hepatocytes cells were seeded on glass coverslips and growth until 70–80% confluence. At this confluency level, cells were either treated with HgCl_2_ diluted in the complete cell culture medium at different concentrations 1, 5 or 10 µM for 1 h at 37° or maintained under control conditions (untreated cells). Afterward, cells were fixed in 95% ethanol/5% glacial acetic acid and post-fixed in Carnoy’s solution (absolute ethanol: Glacial acetic acid 3:1 (vol/vol)) for 30 min. Subsequently, cells were hydrated through graded alcohols to dH_2_O. A solution of 0.66% gelatin in dH_2_O was prepared, to which formic acid was added at a final 0.33% concentration. This solution was pre-warmed at 37 °C and silver nitrate was dissolved in the gelatin–formic acid solution at a final 33% concentration. The slides covered with the fixed hepatocytes were immersed in the silver nitrate-formic acid-gelatin solution and put in an incubator at 37 °C for 25 min. Afterwards, the solution was poured off and the slides were washed in several baths of dH_2_O. Cells were then treated with 5% sodium thiosulfate solution, prepared extemporaneously, for 7 min and rinsed twice in dH_2_O. Coverslips were finally mounted using 90% glycerol in PBS 1X pH 7.2–7.4 and imaged using an Olympus BX51 microscope. Images were submitted to morphometric analyses using the software Fiji (RRID:SCR_002285). We measured the nucleolar organizer regions (NORs) area of 50 nuclei per sample. Statistical significance was evaluated by ANOVA test. *P* < 0.05 was considered statistically significant.

### RT-qPCR

5 million AML12 cells for each condition were cultured and collected as previously described. RNA was extracted from cell pellet using TriZol Ultra Pure Invitrogen kit and then subjected to DNase treatment using DNase I, RNase-free (Thermo Fisher Scientific, Milan, Italy), (~ 1500 ng per sample were treated).

 ~ 800 ng per sample were retrotranscribed using the RevertAid First Strand cDNA Synthesis Kit (Thermo Fisher Scientific, Milan, Italy) according to the manufacturer's suggestions.

qRT‐PCR was carried out with the Maxima SYBR Green qPCR Master Mix (2X; Thermo Fisher Scientific) according to supplier’s indications, using a Rotor‐Gene 6000 PCR apparatus (Corbett Robotics Pty Ltd, Brisbane, Queensland Australia). Amplification conditions were as follows: denaturation at 95 °C for 10 min, and 45 cycles of 95 °C for 15 s and 60 °C for 30 s, final extension at 72 °C for 30 s. Oligonucleotide primers are listed in the Table EMS_2 in the Supplementary information and were designed using Primer3 (RRID:SCR_003139) and further verified with Integrated DNA Technologies OligoAnalyzer (RRID:SCR_001363).

B2m and Tbp were used as reference genes to normalize target genes quantification, as they were validated by (Gong et al. 2016).

### SDS‐PAGE and Western Blotting

For Western‐blot analysis, 5 million AML12 cells for each condition were harvested by trypsinization and centrifuged at 1,200 rpm for 10 min. The cells were lysed adding a buffer (50 mM Tris‐HCl pH 7.5, 100 mM NaCl, 300 mM sucrose, 3 mM MgCl_2_) containing 1 μM leupeptin, 2 μg/mL aprotinin, 1 μg/mL pepstatin, 1 mM phenylmethylsulfonyl fluoride (PMSF) and phosphatase inhibitors. After lysis on ice, homogenates were obtained by passing them through a blunt 20‐gauge needle fitted to a syringe 5 times and all the samples were centrifugated at 3000 rpm for 10 min. Supernatants were removed and each pellet of nuclei was resuspended in 50 μl of RIPA Buffer (50 mM Tris‐HCl pH 7.4, 150 mM NaCl, 5 mM EDTA, 10% glycerol, 1% NP40 buffer, 1 μM leupeptin, 2 μg/mL aprotinin, 1 μg/mL pepstatin and 1 mM PMSF). The protein content of each sample was measured by the BCA protein assay. Western blots were carried out by standard procedures: 20 µg of proteins were separated on 8 or 18% acrylamide/bis‐ acrylamide SDS‐PAGE, transferred onto a nitrocellulose membrane (Millipore, Billerica, MA, USA), probed with the appropriated antibodies and visualized using ECL detection system (Millipore). Protein levels were quantified by densitometry of immunoblots using Scion Image software (Scion Corp., Frederick, MD, USA). The primary antibodies used are listed in the table EMS_3 in the Supplementary information. IgG HRP anti‐rabbit (catalog number #7074) and anti‐mouse (catalog number #7076) conjugated secondary antibodies (purchased by Cell Signaling Technology, Danvers, MA, USA) were diluted 1:8,000. Statistical significance was evaluated by ANOVA test. *P* < 0.05 was considered statistically significant.

## Results

### Heterochromatin regress and assume loose conformation after mercury chloride exposure

The extent of chromatin condensation is an index of gene expression and in turn reflects cell metabolic rate (Knaap and van der Verrijzer 2016). Highly active cells like tumor cells show loose chromatin conformation, while cells with slow or arrested metabolism such as stably differentiated cells tend to show larger blocks of heterochromatin (Beagrie and Pombo 2017).

**Fig. 1:**
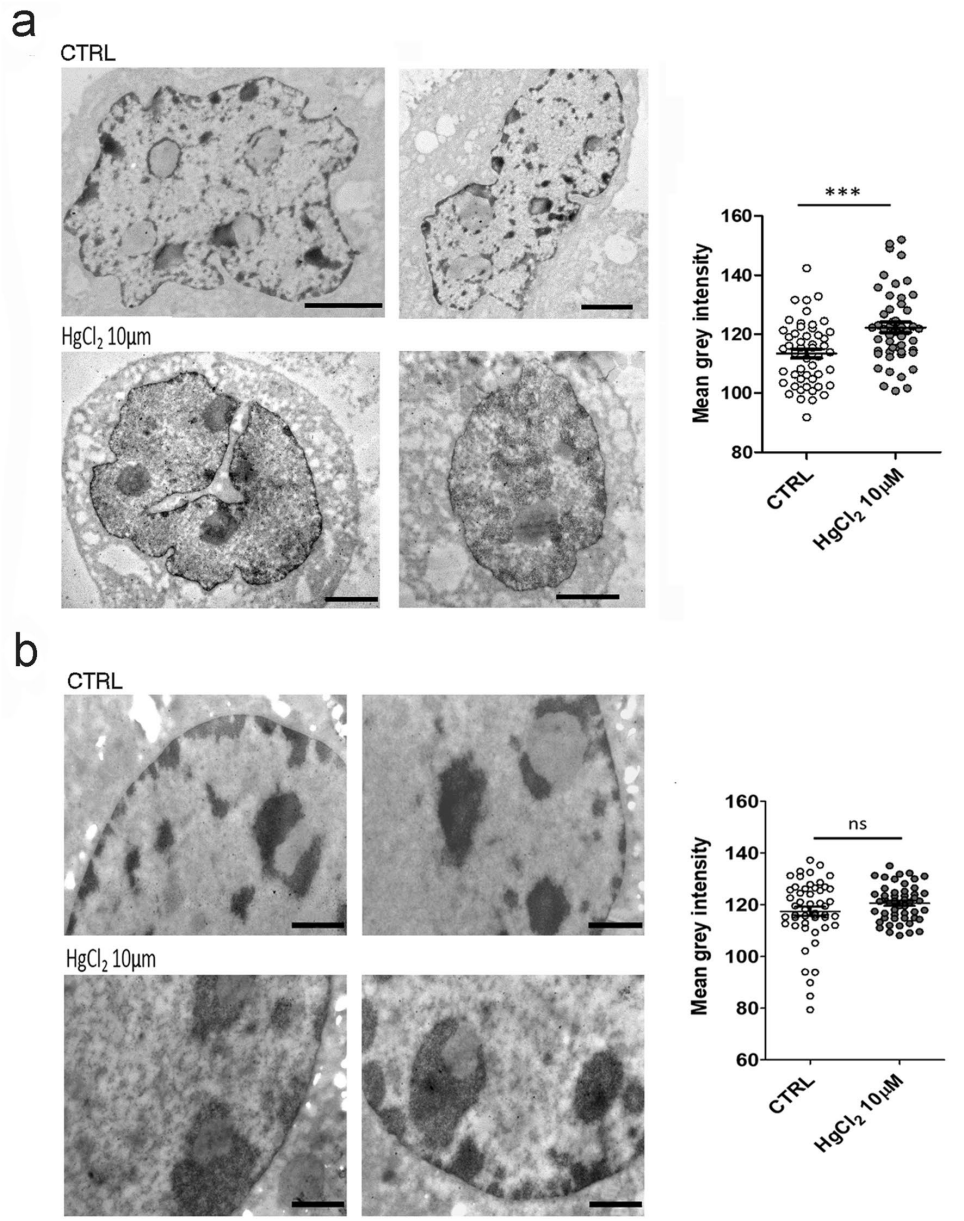
Osmium ammine staining revealed different heterochromatin morphology and organization in control (top) and after HgCl_2_ treatment (bottom) in cell culture hepatocytes, bars: 2 µm (**a**) and in liver tissue, bars: 1 µm (**b**). The graphs show the heterochromatin mean grey intensity ± SEM. Statistical significance was evaluated using unpaired Student’s t test

To investigate in detail the heterochromatin degree of condensation and morphology after mercury chloride treatment, we stained the hepatocytes and liver tissue samples with osmium ammine. This staining allows the specific detection of DNA in the cell nucleus, therefore nuclear regions characterized by high DNA concentration like heterochromatin, appear highly contrasted on a lighter background (Biggiogera et al. 1996). It can be appreciated from the micrographs in Fig. 1a that in cells exposed to mercury chloride heterochromatin domains appear reduced and adopt less dense conformation. To quantify the extent of heterochromatin decondensation, we analysed the mean grey value in the heterochromatin domains, sampling 50 heterochromatin areas per condition, and comparing untreated hepatocytes to those exposed to mercury chloride.

For hepatocytes in cell culture, we can clearly observe on average that the heterochromatin areas selected are lighter and so less dense in treated cells compared to the control (Fig. 1a). However, this effect was not significantly detected through these measurements in hepatic tissue (Fig. 1b).

We further performed Hoechst staining to estimate the relative heterochromatin amount comparing hepatocytes exposed to the toxicity of HgCl_2_ to the untreated control.

When bound to DNA Hoechst fluorescence increases many times (Latt and Stetten 1976). Since the nuclear areas where chromatin has a tightly packaged conformation, the heterochromatin domains, contain locally a major density of DNA, they result in brighter Hoechst staining.

By measuring the total area of the brighter fluorescent dots in each nucleus, applying a threshold, using Fiji software, we detected a decrease in the heterochromatin domains in cells and hepatic tissue exposed to 10 µM HgCl_2_ for 1 h, both in treated hepatocytes from cell culture (Supplementary Fig. ESM_1a) and treated liver tissue (Supplementary Fig. ESM_1b). These results indicated that mercury chloride induces chromatin decondensation or the regress of the tightly package heterochromatin domains in the nucleus.

### Mercury chloride modifies some epigenetic features of heterochromatin

HPTMs play important role in chromatin packaging. Based on the type of specific chemical group added to the histone tail, they can attract or repulse specific proteins or non-coding RNAs which contribute to the formation of higher-order chromatin structures (Taylor and Young 2021).

Considering the effects of mercury on chromatin condensation, we analysed the quantity and the distributions of three epigenetics markers associated to heterochromatin formation and gene silencing: H3K9me3, H3K27me3 and H4K20me3. H3K9me3 is associated with constitutive heterochromatin domains (Nehmé et al. 2015), while H3K27me3 is considered a marker of facultative heterochromatin, so it is more prone to be established or erased in response to different stimuli (Trojer and Reinberg 2007).

H4K20me3 is often found in silenced repeated sequences and transposable elements (Nelson 2016).

Moreover, we quantified the HPTM H3K4me3, which is found in the actively transcribed genes (Howe et al. 2017).

Immunohistochemistry at TEM allowed us to visualize the localization and distribution of these HPTMs in great detail in the nucleoplasm.

**Fig. 2:**
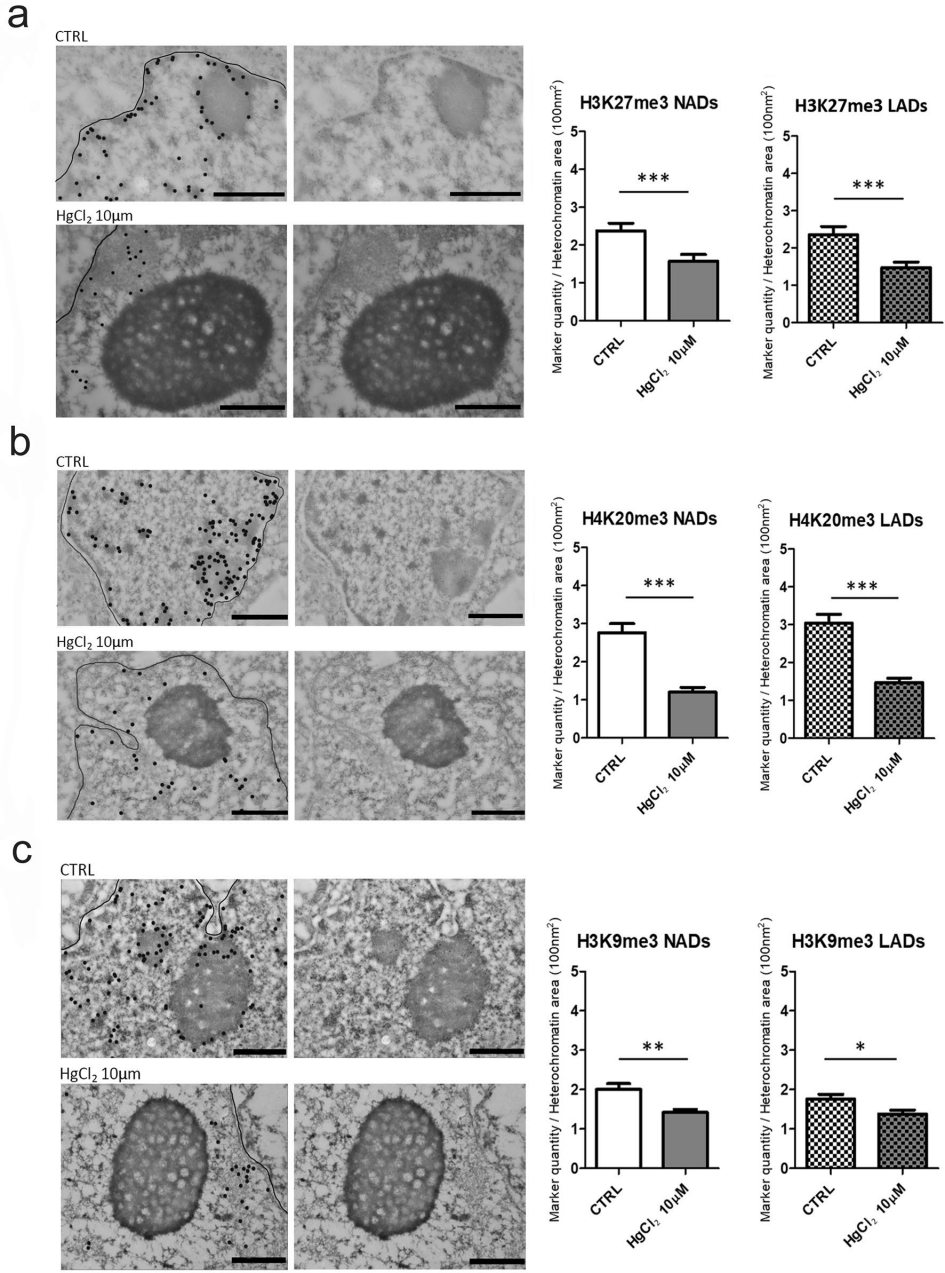
Immunocytochemistry at TEM of H3K27me3, H4K20me3 and H3K9me3. The micrographs show the localizations of H3K27me3 (**a**), H4K20me3 (**b**) and H3K9me3 (**c**) in mouse hepatocytes in culture, which are highlighted by black dots, the line stands for nuclear envelope (left). The corresponding original images are shown on the right. Bars: 1 µm. Histograms show the mean ± SEM quantity of the HPTMs density per heterochromatin area in NADs and LADs for control and HgCl_2_ treated cells. Statistical significance was evaluated using unpaired Student’s t test

**Fig. 3:**
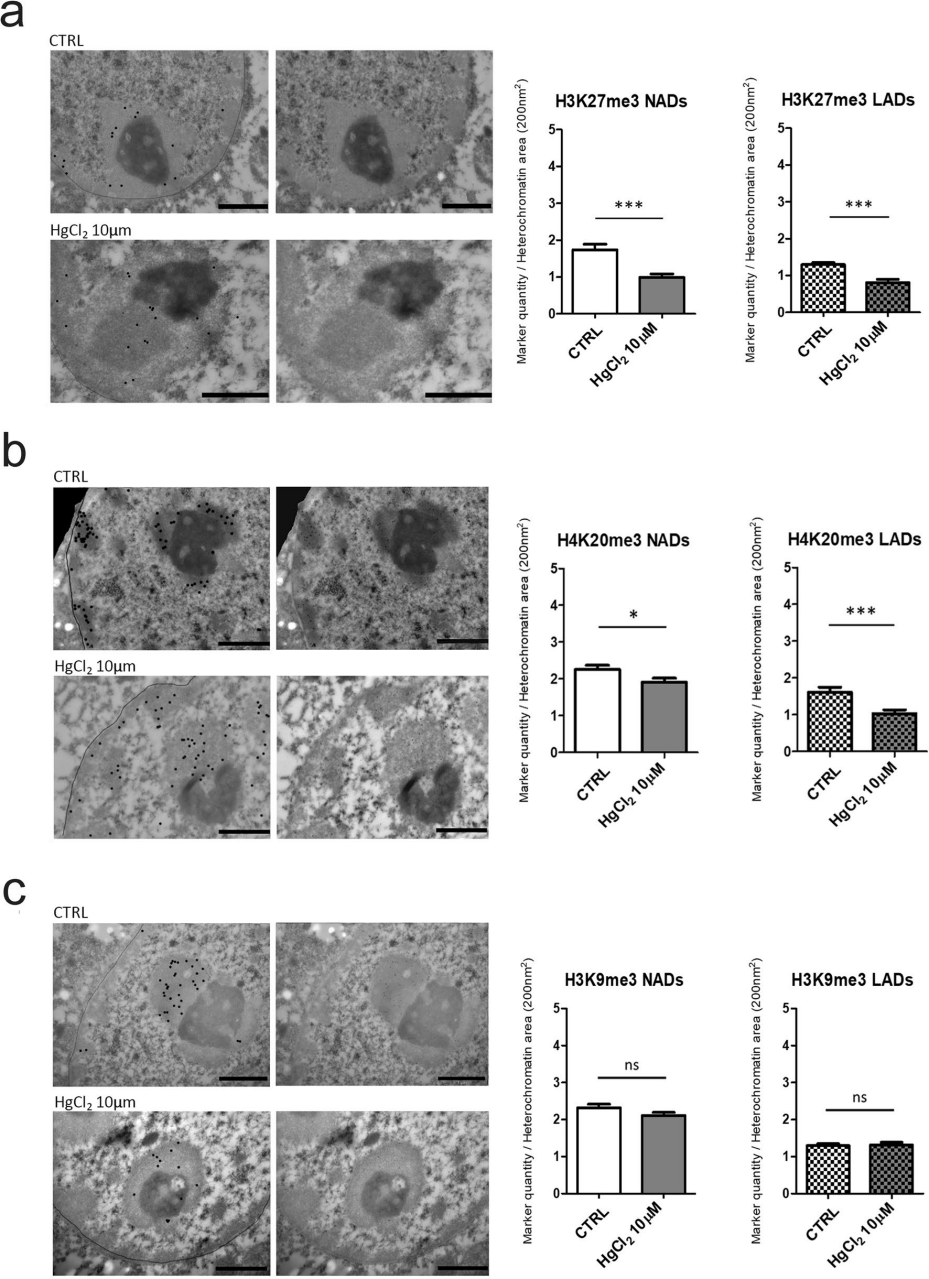
Immunohistochemistry at TEM of H3K27me3 (**a**), H4K20me3 (**b**) and H3K9me3 (**c**). The micrographs show the localizations of H3K27me3 (**a**), H4K20me3 (**b**) and H3K9me3 (**c**) in mouse hepatocytes nuclei from liver tissue, which are highlighted by black dots, the line stands for nuclear envelope (left). The corresponding original images are shown on the right. Bars: 1 µm. Histograms show the mean ± SEM quantity of the HPTMs density per heterochromatin area in NADs and in LADs for control and HgCl_2_ treated cells. Statistical significance was evaluated using unpaired Student’s t test

H3K27me3, H4K20me3 and H3K9me3 epigenetic marks as expected fall all in the heterochromatin domains, which can be distinguished from euchromatin thanks to the EDTA regressive staining. HPTM localizations are indicated by dark spots of 12 nm in the original micrographs on the right and highlighted by bigger black dots to facilitate the visualization on the left in Figs. 2a, b, c and 3a, b, c. By measuring the quantity of these three HPTMs in nucleolus associated domains (NADs) and lamina associated domains (LADs), we found that HgCl_2_ treatment induces a great decrease of these epigenetics markers compared to the control in all heterochromatin areas. This effect was more pronounced on hepatocytes in cell culture (Fig. 2a, b, c), but also present when treating liver tissue (Fig. 3a, b). The histone marker which was subjected to less or no variation was H3K9me3 in hepatocytes from cell culture and liver tissue respectively (Fig. 2c and 3c). This may be expected, being H3K9me3 an epigenetic modification assumed to be more stable. H3K9me3 is known to be established in the late phase of cells differentiation to permanently silence large blocks of repeated sequences, such us centromere and telomere, who were reported to frequently associate with nucleoli forming part of the NADs (Dillinger et al. 2017). The highly significant decrease in H327me3 and H4K20me3 upon HgCl_2_ treatment suggests activation of gene expression.

To confirm these trends observed by immunocytochemistry at TEM, we decided to analyse the expression of the methyltransferases majorly implicated in the establishment of these three HPTMs and to run a western blot analysis to further quantify the different HPTMs protein levels.

**Fig. 4:**
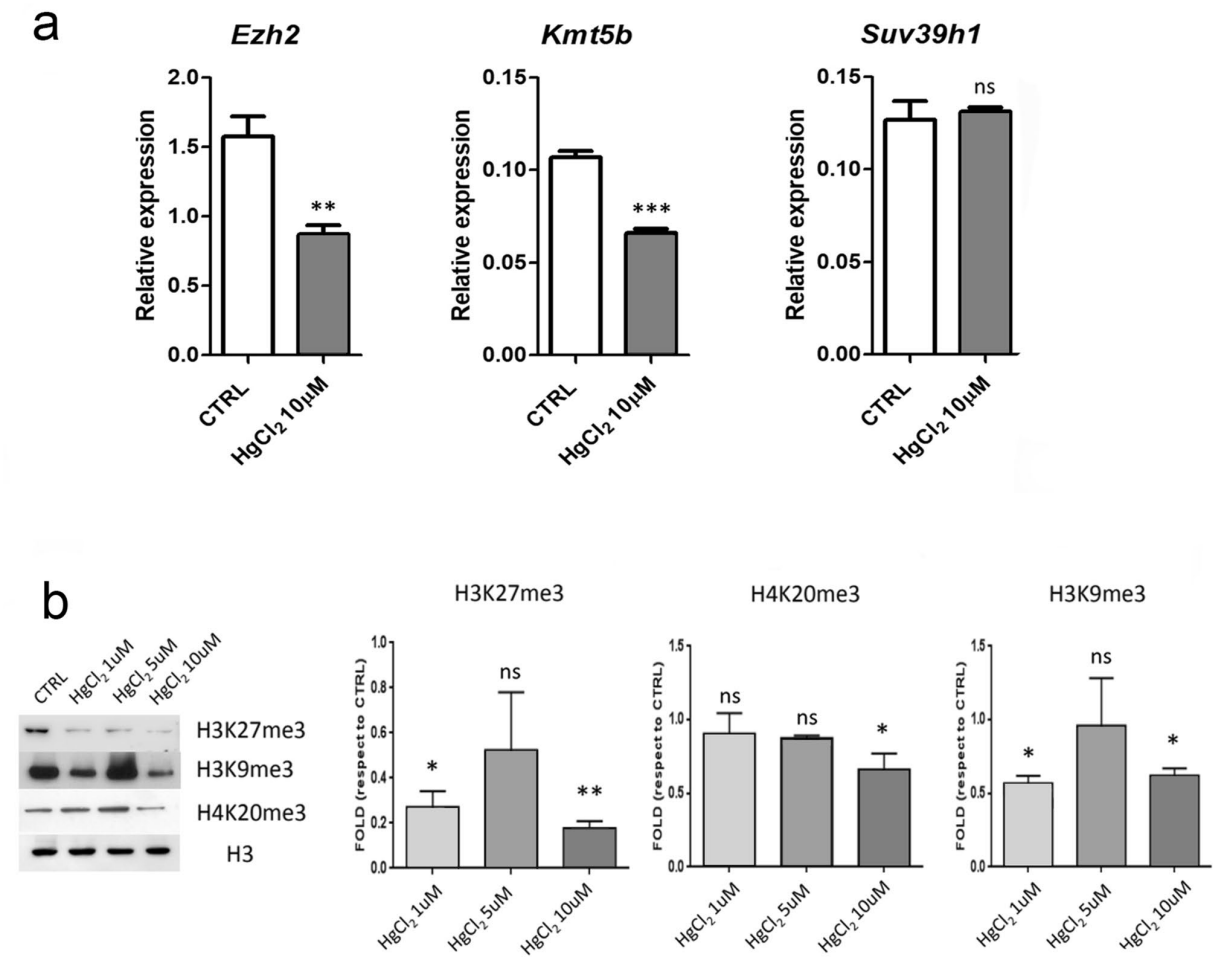
**a** RT-qPCR analysis to quantify the expression of the histone methyltransferases Ezh2, Kmt5b and Suv39h1. Histograms show the mean relative expression ± SD of the gene coding for Ezh2 Kmt5b and Suv39h1 which are implicated in the establishment of H3K27me3, H4K20me3 and H3K9me3 respectively. Statistical significance was evaluated using Student’s t test. **b** Western blot analysis to quantify H3K27me3, H4K20me3 and H3K9me3. Histograms show the mean ± SD of the fold change protein quantity relative to the control. Histone H3 was used as a loading control. Statistical significance was evaluated using One Way ANOVA test

The RT-qPCR analysis revealed that both Kmt5b and Ezh2 expression decrease significantly in hepatocytes exposed to HgCl2 10 µM (Fig. 4a). This is in agreement with the results obtained by immunocytochemistry which suggested a decrease in both products of these methyltransferases. Suv39h1 expression was not significantly distinct between treated and control cells (Fig. 4a). The previous immunocytochemistry analysis also revealed less decrease than the other HPTMs or no significant difference in the quantity of H3K9me3 in hepatocytes from cell culture (Fig. 2c) and liver tissue (Fig. 3c) respectively.

Western blot analysis confirms the trends revealed by the immunocytochemistry at TEM. All three HPTMs associated with the heterochromatin formation decreased significantly after mercury chloride treatment, at least for the highest 10 µM dose (Fig. 4b).

**Fig. 5:**
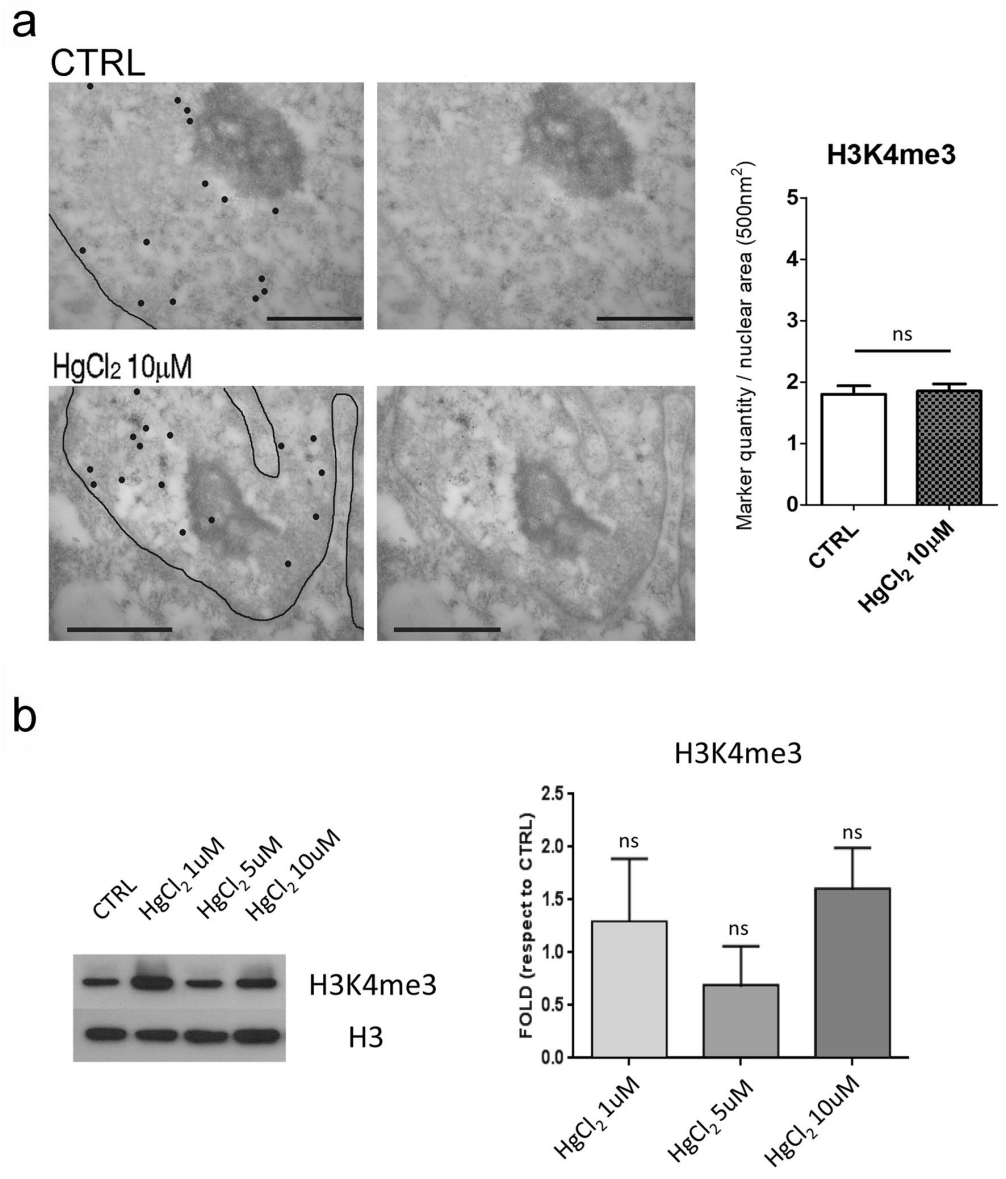
Immunohistochemistry at TEM of H3K4me3. The micrographs show the localizations of H3K4me3 in mouse hepatocytes in culture (**a**), highlighted by black dots; the line stands for nuclear envelope (left). The corresponding original images are shown on the right. Bars: 1 µm. Histograms show the mean ± SEM quantity of the HPTM density per nuclear area. Statistical significance was evaluated using unpaired Student’s t test. **b** Western blot analysis to quantify H3K4me3 protein level. In the histograms are plotted the mean ± SD of the fold change protein quantity relative to the control. Histone H3 was used as a loading control. Statistical significance was evaluated using One Way ANOVA test

Concerning H3K4me3, both immunocytochemistry at TEM in cell culture hepatocytes (Fig. 5a) and western blot (Fig. 5b) revealed no significant difference in the quantity of this HPTM after mercury chloride treatment.

### Nucleolar size and activity increased after HgCl_2_ treatment

Since the nucleolus is the nuclear domain where ribosome biogenesis and other important molecular processes take place (such as pathways that regulate cell proliferation and apoptosis and the formation of telomeres), its size and morphology are subjected to great changes upon different cell stress and stimuli (Yang et al. 2018). Investigating these changes through microscopy and molecular biology could provide important information about cell response after HgCl_2_ cytotoxic damages.

**Fig. 6:**
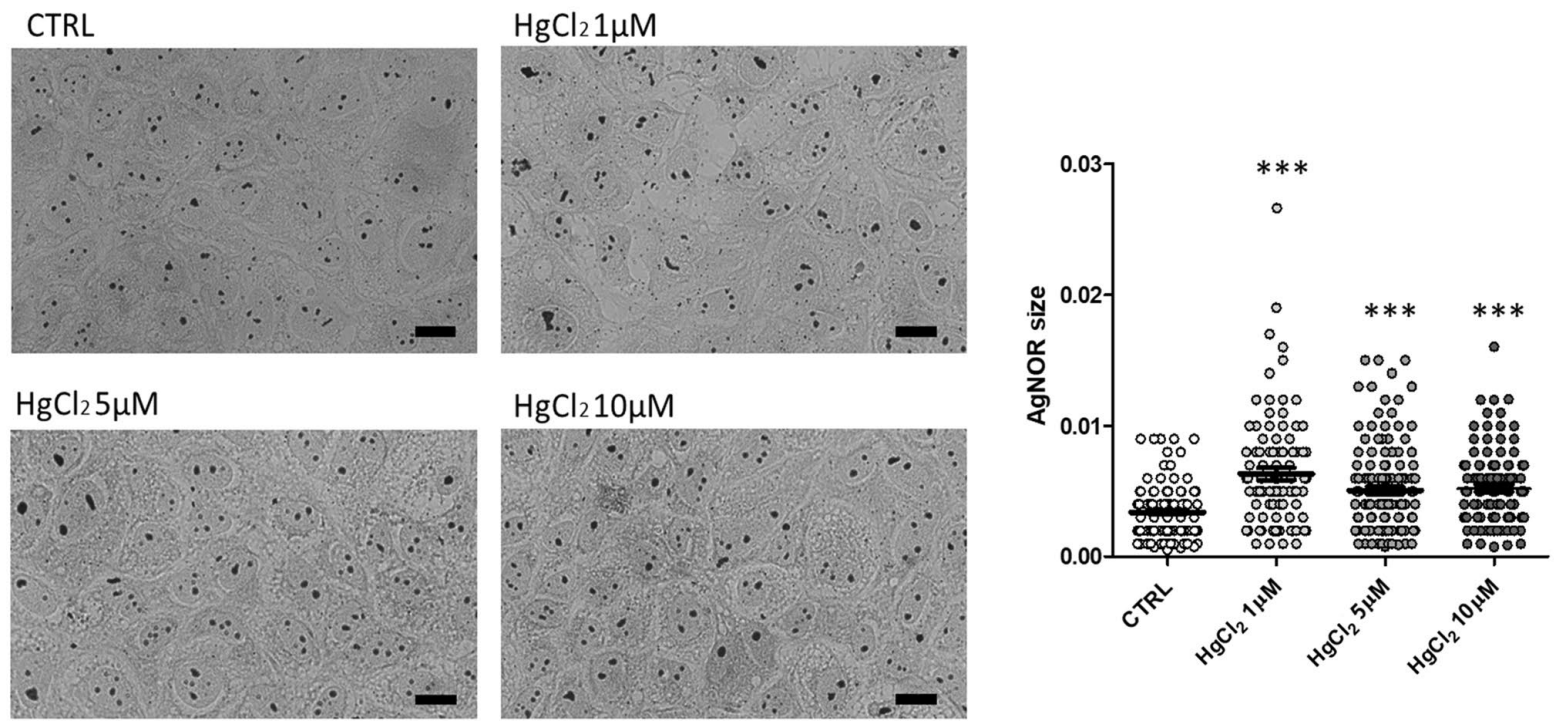
Silver staining of the NORs. AgNOR staining for control and hepatocytes treated with increasing doses of HgCl_2_. Bars: 10 µm. Histograms show the size of the NORs. Bars indicate mean ± SEM. Statistical significance was evaluated using One Way ANOVA test

First of all, we investigated nucleolar activity and morphology through the silver staining of the NORs. These nucleolar components contain a set of argyrophilic proteins, which are selectively stained by silver methods. After staining, they are visualized as black dots localised throughout the nucleolar area, called "AgNORs" (Trerè 2000). We measured the extension of the NORs by comparing control hepatocytes with those treated with increasing doses of HgCl2. NOR areas increase evidently after HgCl_2_ treatment; but the major increase was detected after 1 µM HgCl_2_ exposure, the lowest dose (Fig. 6).

Next, we decided to quantify the extent of ribogenesis by measuring the rRNA expression by RT-qPCR.

**Fig. 7:**
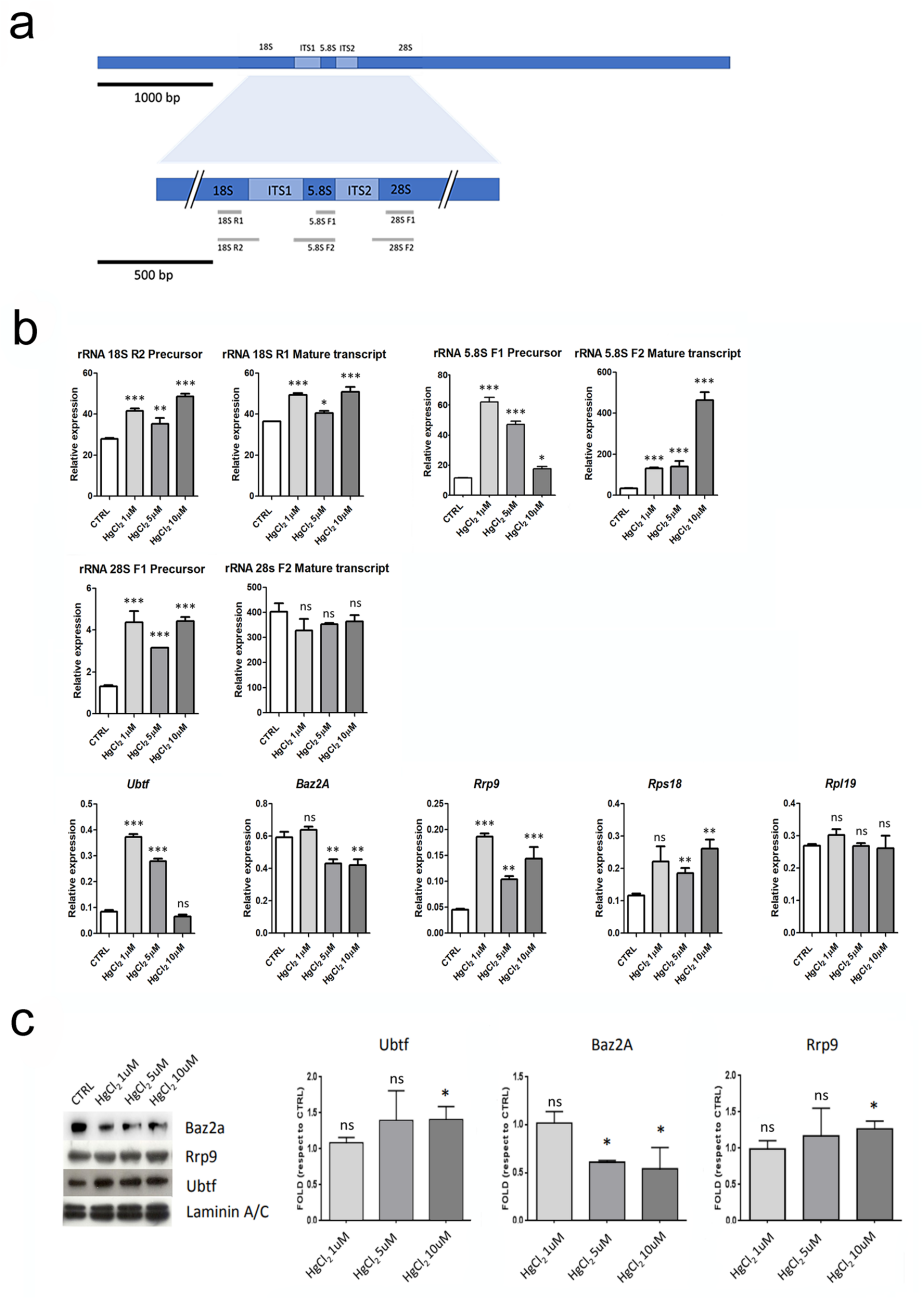
**a** Schematic representation of primers design for rRNAs quantification by RT-qPCR. **b** RT-qPCR analysis to quantify the expression of rRNAs and some proteins involved in the ribogenesis process. In the histograms are plotted the mean ± SD of the relative gene expression. Statistical significance was evaluated using One Way ANOVA test. **c** Western blot analysis to quantify the expression of proteins involved in the ribogenesis process. Histograms show the mean ± SD of the fold change protein quantity relative to the control. Lamin A/C was used as a loading control. Statistical significance was evaluated using One Way ANOVA test

To monitor the trend of the ongoing process of rRNA transcription, we designed primers to amplify not only the final processed rRNA 18S, 5.8S and 28S, but also some corresponding precursors, by using primers matching on the rDNA intergenic sequence just upstream or downstream of the mature transcripts (Fig. 7A; for detailed information about the sequences of the primers see the Supplementary Table EMS_2).

In agreement with the morphological analysis after agNOR staining, RT-qPCR revealed that ribogenesis increases after HgCl_2_ treatment more or less in a dose dependent trend, with some difference considering each transcript. This is interesting, because it could reflect an impact on different moments of ribogenesis. We detected an increase in the precursors for all three transcripts. Specifically, 5.8S majorly increased after hepatocytes treatment with the lowest 1 µM HgCl_2_ dose, while for the mature 5,8S transcript we detected the highest increase after the highest 10 µM HgCl_2_ dose exposure. This may reflect the increase in the speed of the 5,8S rRNA synthesis after 1 µM HgCl_2_ treatment and impairments in this process with the accumulation of the mature 5,8S transcript after 10 µM HgCl_2_ exposure. Concerning the 28S we saw an increase in the precursor, but not in the final transcript product. Both precursor and mature transcripts quantity of 18S, evidently increased after HgCl_2_ treatment (Fig. 7b).

To gain further insights we decided to analyse also the expression of other proteins and transcription factors associated, with different roles, to the ribogenesis process. We analysed the expression of three transcription factor: ubtf, baz2a and rrp9. We detected an increase in both Ubtf (although not for the highest dose) and Rrp9 genes and a decrease in Baz2a expression in treated cells. These results suggest again nucleolar activation after HgCl_2_ exposure (Fig. 7b). Moreover, it is reported that baz2a mediates rDNA gene silencing through the establishment of H3K9me3 and H4K20me3 (Guetg et al. 2010). The decrease in the expression of this gene is also in agreement with the results of immunocytochemistry at transmission electron microscopy, where we detected a decrease in these HPTMs (Fig. 2b, c, 3b).

Finally, as supporting evidence of an increase in ribosome production, we analysed the expression of genes coding for one component of the small ribosomal subunit the rps18 and one protein of the large ribosomal subunit the rpl19. We detected an increase in Rps18 but not in Rpl19 (Fig. 7b). We finally analysed the quantity of Ubtf, Rrp9 and Baz2a proteins by western blot. This analysis further confirmed an increase in Ubtf and Rrp9 proteins and a decrease in Baz2a after mercury chloride treatment, at least for the highest 10 µM dose (Fig. 7c).

## Discussion

Mercury chloride exposure is an important concern due to its high toxicity and wide environmental spread. In this study, we analyzed the effects of this toxicant on the heterochromatin organization and nucleolar activity to understand its detrimental consequences on the primary steps of cell metabolism: modulation of gene expression and ribosome production.

Our data suggest a general cell activation after HgCl_2_ treatment, which is realized by an extensive heterochromatin decondensation, clearly visible by our microscopy approaches and reflected by the quantification of the epigenetic markers associated to heterochromatin.

Specifically, images obtained at TEM by staining the nuclei with osmium ammine showed that HgCl_2_ remodels the heterochromatin which appears more dispersed and less compact. The fluorescent staining by Hoechst provided parallel evidence of the regression of the heterochromatin areas.

Epigenetic modifications influence the nuclear architecture by providing chemical groups that by attraction or repulsion lead to the construction or disassembly of higher-order chromatin structures (Taylor and Young 2021).

An important contribution to reach the HgCl_2_ induced decondensed heterochromatin conformation probably derives from the significant decrease we detected in H3K27me3, H4K20me3 and H3K9me3, epigenetic modifications known to be associated with heterochromatin formation and gene silencing.

Interestingly, our analysis highlights an evident decrease of H3K27me3, which is a marker of facultative heterochromatin and a modest decrease of H3K9me3, considered a signature of constitutive repressed chromatin. We exposed hepatocytes to the toxicant for one hour, in such time probably extensive changes in a heterochromatin marker persistently present on specific heterochromatin portions, possibly constituting the core of tightly close heterochromatin domains may be difficult to imagine. However, the toxic effects of mercury on the nucleus seem to be so powerful to induce evident changes in the general heterochromatin organization. Indeed we detected not only a modest reduction in H3K9me3, but also a significant decrease of H4K20me3, which is usually also associated with constitutive heterochromatin as well. These results suggest that even 1 h of exposure to HgCl_2_ can deeply modify nuclear architecture and gene activity. This heterochromatin derepression seems to be accompanied by nucleolar activation, maybe to rapidly translate the enzymes necessary for detoxification. We saw an increase in the nucleolar activity, as bigger NORs areas, by silver staining. It was also possible to appreciate in the micrographs obtained at TEM that hepatocytes from cell culture exposed to HgCl_2_ show large nucleoli with many fibrillary centers (Fig. 2a, b, c). Moreover, we have analyzed the levels of 18S, 5,8S and 28S rRNAs, one precursor for each of these transcripts and some proteins involved in the ribogenesis, to get insights into the HgCl_2_ effects on the different parts of the ribogenesis, since it is a complex molecular process.

We detected an increase in the 18S and 5.8S transcripts and their corresponding precursors. Furthermore, Ubtf and Rrp9 increased their expressions, while Baz2a expression decreased after HgCl_2_ exposure. The first two enzymes are involved in the activation while the latter in the repression of the rDNA transcription (Chen et al. 2016; Abdel Moneim 2015). All these results indicate the activation of ribogenesis process. However, no increase for the 28S, the last transcribed and processed transcript, and no increase in the expression of the large subunits protein Rpl19 were detected.

This may suggest that HgCl_2_ damage induces nucleolar activation that is effective, especially in the first parts of the ribogenesis. Nevertheless, it should be considered that it may be difficult to highlight changes in the quantity of the mature 28S rRNA after short stimulations, because of the wide accumulation of this transcript in the nucleolus. In fact, 28S is a component of the large ribosomal subunit that lasts in the nucleolus longer than the small subunit which is more rapidly transferred outside the nucleus after its formation (Henras et al. 2014).

Even though this analysis does not provide a direct applicative solution to mercury-induced damages, we believe it is essential to understand its toxic effects on these basic molecular processes of the cell nucleus. Our data could at least lay the ground to develop strategies and approaches for the limitation and treatment of mercury toxicity, to restore the cell to its initial physiological state or bring it to a new healthy equilibrium.

Of note, the chelating agents used to date for medical treatment of mercury toxicity, which form chelation compounds with toxic metal ions that are then eliminated from the body by the excretory system, show themselves some extent of toxicity and are ineffective in repairing tissue damages (Wiggers et al. 2008; Zannino et al. 2021; Abdel Moneim 2015).

## Supplementary Information

Below is the link to the electronic supplementary material.

Supplementary file1 (PDF 675 KB)

## Data Availability

The datasets generated during and/or analysed during the current study are not publicly available, but are available from the corresponding author on reasonable request.

## Abbreviations

ATSDR: Agency for toxic substance and disease registry
ATP: Adenosine triphosphate
GSH: Glutathione
ROS: Reactive oxygen species
MAPK: Mitogen-activated protein kinases
NF-κB: In nuclear factor kappa-B
H3K9me3: Trimethylation on histone H3 on lysine 9
H3K27me3: Trimethylation on histone H3 on lysine 27
H4K20me3: Trimethylation on histone H4 on lysine 20
H3K4me3: Trimethylation on histone H3 on lysine 4
rRNA: Ribosomal RNA
UCE: Upstream control element
R.T.: Room temperature
PBS: Phosphate saline buffer
qRT‐PCR: Quantitative real‐time polymerase chain reaction
TEM: Transmission electron microscopy
DMEM: Dulbecco’s modified eagle medium
NGS: Normal goat serum
dH_2_O: Distilled water
EDTA: Ethylenediaminetetraacetic acid disodium salt solution
HPTM: Histone post-translational modification
NORs: Nucleolar organizer regions
ANOVA: Analysis of variance
CTRL: Control
SEM: Standard error of the mean
NADs: Nucleolus associated domains
LADs: Lamina associated domains
PcG: Polycomb group protein
snoRNP: Nucleolar small nuclear ribonucleoprotein particle
