# Supplementary material for: Mercury chloride alters heterochromatin domain organization and nucleolar activity in mouse liver
Source: Histochem Cell Biol. 2022 Sep 22;159(1):61–76. doi: 10.1007/s00418-022-02151-8 (PMC9899742; doi:10.1007/s00418-022-02151-8)
Supplement: Supplementary file 1 — Supplementary file1 (PDF 675 KB) [file 418_2022_2151_MOESM1_ESM.pdf]

Supplementary information

Mercury Chloride Alters Heterochromatin Domain Organization and Nucleolar Activity in Mouse Liver

Lorena Zannino[1], Andrea Pagano[1], Claudio Casali[1], Monica Oldani[2], Alma Balestrazzi[1], Marco Biggiogera[1]*

1. Department of Biology and Biotechnology 'L. Spallanzani', University of Pavia, 27100, Pavia, Italy;
2. Department of Biology and Biosciences, University of Milano-Bicocca, 20126, Milan, Italy

* Correspondence: lorena.zannino@unipv.it

Table EMS_1 List of the primary antibodies used for immunocytochemistry

Antibody target	Antibody Isotype	Source	Working concentration
Histone H3K4me3 (tri-methyl Lys4)	Rabbit IgG	GeneTex (Cat# GTX128954, RRID:AB_2783841)	1:50
Histone H4K20me3 (tri- methyl Lys20)	Rabbit IgG	GeneTex (Cat# GTX128960, RRID:AB_2885848)	1:100
Histone H3K27me3 (Tri-methyl Lys27)	Rabbit IgG	GeneTex (Cat# GTX121184, RRID:AB_10618572)	1:50
Histone H3K9me3 (Tri-methyl Lys9)	Rabbit IgG	GeneTex (Cat# GTX121677, RRID:AB_10721938)	1:10

Table EMS_2 Sequence of the primers used in this study

Gene	Primer sequence
Ezh2 Fw	TGGAGTTGGTAAATGCTCTTGG
Ezh2 Rev	CGGTGCCCTTATCTGGAAAC
Kmt5B Fw	CTGGAAGAACTGGCTCCTG
Kmt5B Rev	GGATGAGACCCTGGCAAATC
Suv39h1 Fw	AGGGGAGGAAGAAGTGGAAC
Suv39h1 Rev	CCAAGGGCAGGACAAGAAAG
18S FW	CGTTGATTAAGTCCCTGCCC
18S R1	GGTTCACCTACGGAAACCTTG
18S R2	CGCTCCTCCACAGTCTCC
5.8S F1	CGCTCACACCCGAAATACCGA
5.8S F2	GGTGGATCACTCGGCTCGTG
5.8S Rev	CAACCGACGCTCAGACAGGC
28S F1	CGTGTCCCCCCTTTCTGAC
28S F2	CGACCTCAGATCAGACGTG
28S Rev	GTCTTCCGTACGCCACATTT
Ubtf Fw	TGAGTCCAGCAGTGAAGATGA
Ubtf Rev	AGAATCCGAAGAGTCCCCTG
Baz2a Fw	GAGGAGGAGAGAGAGGTGG
Baz2a Rev	TGGGAAGGCGGGAATAAAC
Rrp9	GTGAGTTCTTCGGGGTAGC
Rrp9	CTCTTCATTCATTTTGCCGCC
Rps18 Fw	CGGAAAATAGCCTTCGCCAT
Rps18 Rev	ATCACTCGCTCCACCTCATC
Rpl19 Fw	AGACCAAGGAAGCACGAAAG
Rpl19 Rev	AAGAGGGCAACAGACAAAGG
Cdkn1a Fw	GATGGTGTCTTGGGGGC
Cdkn1a Rev	GGATGTTTGGGGCTGGAG
Tp53 Fw	GCTTTGAGGTTCGTGTTTGTGC
Tp53 Rev	CTTTTGCGGGGGAGAGGC

Table EMS_3 List of the primary antibodies used for Western blot analysis

Antibody target	Antibody Isotype	Source	Working concentration
Histone H3K4me3 (tri- methyl Lys4)	Rabbit IgG	GeneTex (Cat# GTX128954, RRID:AB_2783841)	1:500
Histone H4K20me3 (tri- methyl Lys20)	Rabbit IgG	GeneTex (Cat# GTX128960, RRID:AB_2885848)	1:500
Histone H3K27me3 (Tri-methyl Lys27)	Rabbit IgG	GeneTex (Cat# GTX121184, RRID:AB_10618572)	1:500
Histone H3K9me3 (Tri-methyl Lys9)	Rabbit IgG	GeneTex (Cat# GTX121677, RRID:AB_10721938)	1:500
Lamin A/C	Mouse IgG	Santa Cruz Biotechnology (Cat# sc-7292, RRID:AB_627875)	1:500
UBF	Mouse IgG	Santa Cruz Biotechnology (Cat# sc-13125, RRID:AB_671403)	1:500
Baz2A (TIP5)	Rabbit IgG	Santa Cruz Biotechnology (Cat# 49-1037, RRID:AB_2533886)	1:500
RRP9	Rabbit IgG	Biorbyt orb185971	1:500

a

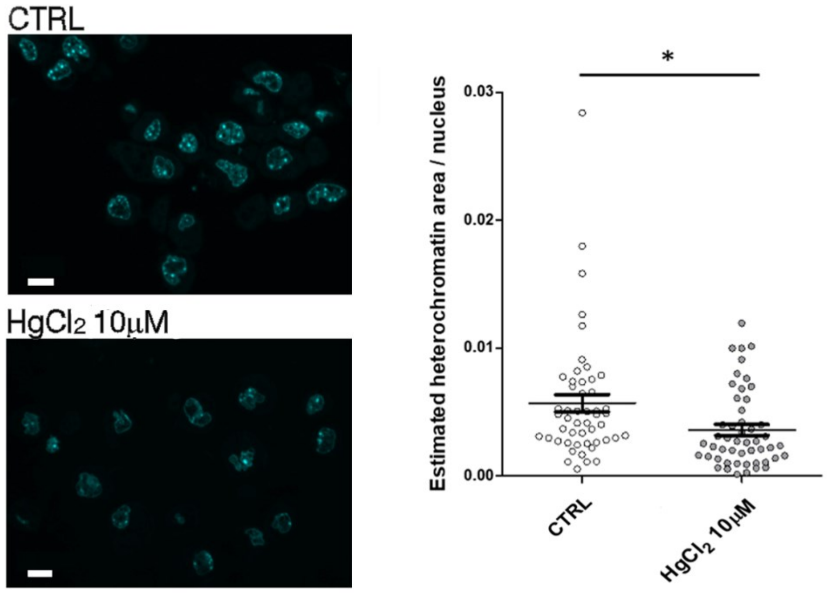

b

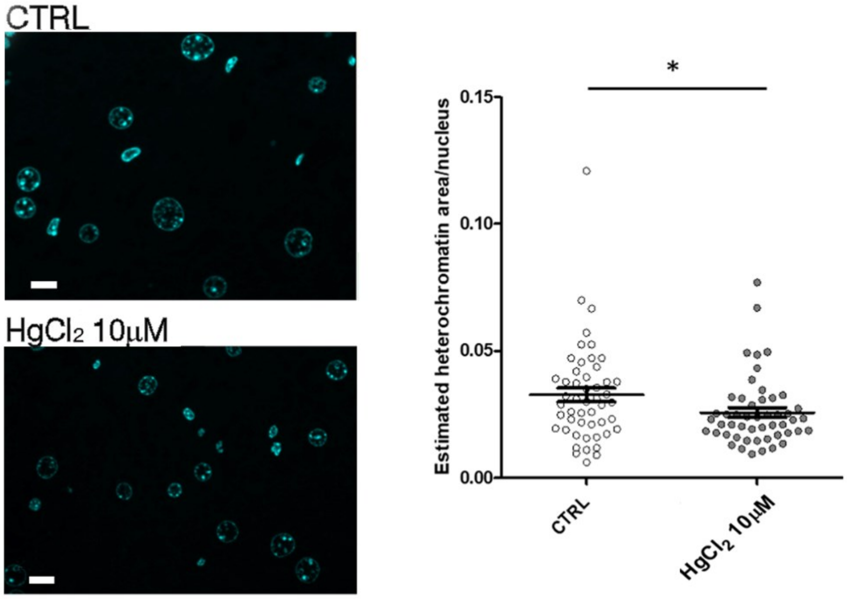

Fig. ESM_1 Hoechst staining to estimate the relative heterochromatin amount in cell-cultured hepatocytes (a) and liver tissue (b). Images show Hoechst fluorescence in mouse hepatocytes nuclei, in control (top) and after $HgCl_2$ treatment (bottom). Bar: 10μm. The graphs show the heterochromatin area per nucleus estimated as the sum of integrated fluorescence intensity of fluorescent dots inside each nucleus. Statistical significance was evaluated using unpaired Student's t test. Bar: mean ± SEM
